# Accuracy of WHO immunological criteria in identifying virological failure among HIV-infected adults on First line antiretroviral therapy in Mwanza, North-western Tanzania

**DOI:** 10.1186/s13104-016-2334-6

**Published:** 2017-01-17

**Authors:** Daniel W. Gunda, Benson R. Kidenya, Stephen E. Mshana, Semvua B. Kilonzo, Bonaventura C. T. Mpondo

**Affiliations:** 1 Department of Medicine, Weill Bugando School of Medicine, Mwanza, Tanzania; 2 Department of Biochemistry and Molecular Biology, Weill Bugando School of Medicine, Mwanza, Tanzania; 3 Department of Microbiology/Immunology, Weill Bugando School of Medicine, Mwanza, Tanzania; 4 Department of Medicine, College of Health Sciences, The University of Dodoma, P.O Box 395, Dodoma, Tanzania

**Keywords:** HIV/AIDS, Antiretroviral therapy, Treatment failure, Immunological monitoring

## Abstract

**Background:**

Optimal HIV treatment monitoring remains a big challenge in resource limited settings. Guidelines recommend the use of clinical and immunological criteria in resource limited settings due to unavailability of viral load monitoring; however their utility is questionable. This study aimed at assessing the accuracy of immunological criteria in detecting treatment failure among HIV infected Tanzanian adults receiving first line ART.

**Methods:**

A clinic based cross sectional study was conducted between February and July 2011 at Bugando Medical centre (BMC) HIV care and treatment clinic (CTC) involving HIV infected patients aged 18 years and above, receiving first line ART; followed up for at least 1 year. Viral load was tested for every enrolled patient. Standard WHO criteria were used to define immunological failure. Virological failure was defined as one viral load measurement of >5000 copies/ml and was used as a gold standard. A 2 × 2 table was used to assess the accuracy of immunological criteria in detecting treatment failure.

**Results:**

A total of 274 HIV-infected adults were enrolled into the study. Out of these, 65.7% were females, the median age was 39 years (IQR 33–45), the median BMI 21.9 kg/m^2^ (IQR 19.7–24.0). Out of the 274 study participants 156 (56.9%) had immunological failure. Only 60 of the study participants (21.9%) had viral load >5000. Only 42 patients (70%) were found to have both immunological failure and virological failure. The sensitivity of immunological criteria in detecting treatment failure was 70%, specificity 46.7%, positive predictive and negative predictive values of 26.9 and 84.7% respectively.

**Conclusion:**

WHO immunological criteria have low sensitivity and positive predictive value for detecting treatment failure. Relying on CD4 counts for treatment monitoring would therefore lead to misclassifications of treatment failure that could result into unnecessary or delayed switch to second line ART. Access to viral load monitoring is important to avoid these misclassifications.

## Background

HIV remains a public health concern in sub-Saharan Africa despite the epidemic being on its 3rd decade. Estimates show that up to 70% of HIV cases are still found in sub-Saharan Africa [1]. Efforts from the World Health Organization (WHO) and the Joint United Nations Programme on HIV/AIDS (UNAIDS) have ensured rapid scaling up of antiretroviral therapy in resource limited settings (RLS) [2]. In 2011, it was estimated that 6.6 million people were receiving ART. Despite the reduction in mortality following the scaling up of antiretroviral therapy (ART) use, optimal monitoring for treatment failure remains a big challenge in RLS.

Guidelines for the use of ART in resource limited settings (RLS) where viral load monitoring is not routinely available recommend the use of CD4 count and clinical monitoring to diagnose treatment failure [3]. WHO guidelines for a public health approach to ART define immunological failure as: the fall off CD4 count to below baseline in the absence of concurrent infections, a fall of more than 50% from the peak value or persistent CD4 below 100 cells/mm^3^ while on treatment [3]. Viral load monitoring which is the gold standard in detecting treatment failure is not readily available in RLS because of the cost and technical requirements of the assay.

Several studies have found low sensitivity and positive predictive value of the WHO immunological criteria on predicting treatment failure [4–7]. This leads to misclassifications and unnecessary switching off to second line ART in patients with adequate virological suppression. However, there are no studies assessing the sensitivity of the immunological criteria from Tanzania. This study therefore aimed at assessing the accuracy of WHO immunological criteria in detecting ART treatment failure in HIV-infected adults taking first line ART.

## Methods

### Study design

This was a clinic based cross sectional study done at Bugando Medical Centre (BMC) HIV care and treatment clinic (CTC) between February and July 2011.

### Study setting

The study was conducted at Bugando care and treatment centre (CTC) in Mwanza, Tanzania. Bugando is a tertiary and teaching hospital for the Lake Zone of Tanzania. The hospital serves around 13 million people from 6 regions of the Lake Zone, which are Mwanza, Kagera, Shinyanga, Tabora, Mara and Kigoma. The hospital runs both inpatient and outpatient treatment activities, with an approximate bed capacity of 900. CTC activities is one of the core part of outpatient activities, which started in 2004, and currently it serves more than ten thousand patients, of whom about 5000 are on ARTs. More than two-thirds of these patients are on first line regimens and the rest are on the second line regime.

### Study participants

The study involved 274 adult HIV-infected patients aged 18 years and above enrolled to HIV care and initiated on first line ART, with a minimum follow up of 1-year. Patients who were critically ill, with concurrent infections, and those on second line were excluded from the study.

### Sample size and sampling

Sample size was estimated using Kish and Leslie formula. A minimum sample size was 220 patients obtained by assuming that 50% of the patients on first line ART will have virological failure. Patients fulfilling the inclusion criteria were serially enrolled until sample size was reached.

### Data collection and laboratory analysis

The adult HIV patients on first line ART with a minimum follow up of 1 year (12-months) were identified from daily CTC listing at Bugando on routine basis and they were invited to participate in this study. These patients are usually followed up monthly or after every 2-month depending on their clinical status. CD4 measurements are usually routinely performed after every 6-month. After giving consent a structured questionnaire was used to collect information regarding, demographic data, date of diagnosis of HIV, date of ART initiation and regime, compliance level which was assessed using patient response method, BMI, TB status, co morbidities, co medications, CD4, VL, ART serum levels, and other routine laboratory. For each patient enrolled into the study, 1.5 ml of blood was collected in an EDTA bottle for viral load. This sample was also centrifuged in the ICU side lab, at 3000 rpm to obtain plasma which was again transferred into cryovials and sent to BMC main lab for viral load using FACS—calibur analyzers according to manufacturer’s guidelines.

### Statistical analysis

The data was entered, verified and a cleaned, using Microsoft excel spread and the data analysis was done using STATA version 14 (College Station, Texas). Continuous variables were summarized by medians and interquartile ranges (IQRs) and categorical variables were summarized by frequency and percentage. A 2 × 2 table was used to assess the accuracy of the immunological criteria for detecting treatment failure using viral load as the gold standard.

### Definitions of treatment failure

Definitions for immunological failure were derived from the WHO guideline for this analysis [3]. A participant was therefore considered to have immunological failure if one of the following criteria was met: (1) fall of CD4 count to pre-therapy baseline or below, (2) ≥50% fall of absolute CD4 count from the on-treatment peak value or (3) persistent CD4 levels below 100 cells/mm^3^; all in the absence of concurrent infections. Virological failure was defined as one viral load measurement of >5000 copies/ml.

## Ethics and consent

### Ethics approval

This study was approved by Bugando Medical centre (BMC)/Catholic University of Health and Allied Sciences (CUHAS) joint research and ethical committee. Further approval was sought from the department of Internal medicine, Bugando Medical centre.

### Consent to participate

Informed consent to participate in the study was obtained from all study participants. The study enrolled only patients who provided written consent. All patients with immunological failure were switched into second line of ARV following failure of improvement of their CD4 two weeks of intensified adherence and in some with virological proof of ARV treatment failure as per existing treatment guidelines. Patients who declined consent were not denied of their services.

## Results

A total of 274 HIV-infected adults were enrolled into the study. Out of the study participants, 65.7% were females, the median age was 39 years (IQR 33–45), the median BMI 21.9 kg/m^2^ (IQR 19.7–24.0) (Table 1). Majority of the study participants (47.8%) presented with WHO clinical stage 3, the median baseline CD4 count being 139.5 cells/mm^3^ (IQR 60–210). Most of the study participants (97.4%) had adherence level of >95%.

**Table 1:** Baseline characteristics of HIV-infected adults attending BMC-CTC (n = 274)

Variable	Number (%) or median (IQR)
Age (years)	39 (33–45)
Gender	
Female	178 (65.7)
Males	93 (34.3)
Baseline CD4 (cells/mm^3^)	139.5 [IQR 60–210]
WHO stage	
Stage 1	11 (4.0)
Stage 2	66 (24.1)
Stage 3	131 (47.8)
Stage 4	66 (24.1)
BMI (kg/m^2^)	21.9 (19.7–24.0)
ARV regimes	
Stavudine based	34 (12.4)
Zidovudine based	129 (47.1)
Tenofovir based	111 (40.5)
Adherence level (%)	
>95	267 (97.4)
<95	7 (2.6)

Based on the WHO criteria for immunological failure, out of the 274 study participants 156 (56.9%) met the criteria. Only 60 of the study participants (21.9%) had viral load >5000. Only 42 participants out of the 60 with confirmed virological failure (70%) were found to have both immunological failure and virological failure. The sensitivity of immunological criteria in detecting treatment failure was therefore 70% with specificity and positive predictive values of 46.7 and 26.9% respectively. The negative predictive value was found to be 84.7% (Table 2). 30% of the study participants who fulfilled the criteria for virological failure did not fulfill any of the criteria for immunological failure. Out of the 156 patients who fulfilled criteria for immunological failure, 73.1% were virologically suppressed.

**Table 2:** Test characteristics of WHO immunological criteria to detect treatment failure among HIV infected adults (n = 274)

Immunological failure	Virological failure		
	Yes	No	Total
Yes	42	114	156
No	18	100	118
Total	60	214	274

*Sensitivity* 70%, *specificity* 46.7%, *positive predictive value* 26.9%, *negative predictive value* 84.7%

## Discussion

In this study, the performance of WHO immunological criteria in detecting treatment failure was poor. Despite a relatively high NPV (84.7%), the PPV was low (26.9%). Using the WHO immunological criteria would miss up to 30% of the patients who were truly having treatment failure while it would misclassify as treatment failure 73.1% virologically suppressed patients. In this cohort, 21.9% of the participants had virological failure.

These findings are consistent with studies elsewhere in resource limited settings. A study analyzing data from 10 programmes in Africa and south America found that the PPV ranged from 10 to 30% with a relatively higher NPV [4]. Another study done in Kenya found that immunological criteria had a PPV of 24.5% with a 95.2% NPV. Another study done in Nigeria found a PPV of 39.2% and a NPV of 86.7% more or less similar to our study [8]. Another study done in India found that WHO immunological criteria had a PPV of 34.2% [9].

The low PPV found in this study means that only a few of the patients that were identified as having treatment failure by the immunological criteria were actually having treatment failure. The false positive rate of immunological criteria for this study was 73.1%. This is comparable to a study done in Nigeria which found the false positive rate to be around 60.8%. The low specificities and PPV of immunological criteria found in our study, that is similar to many other studies in Africa and other RLS mean that patients with adequate viral suppression are at risk of being classified as having treatment failure and switched to second line. Monitoring treatment failure using immunological and/or clinical criteria is associated with the emergence of nucleoside reverse transcriptase inhibitor and non-nucleoside reverse transcriptase inhibitor resistance [10]. Based on immunological criteria in this study, 30% of the patients would have been classified as not failing, and therefore not switched to second line. Failure to switch a failing regimen has been shown to result into accumulation of mutations that will limit future treatment options [9, 11].

The sensitivity in this study was relatively higher compared to other studies. This could be explained by the cut off used in this study (>5000 copies/ml) which has been suggested by WHO to increase sensitivity in resource limited settings [3]. Another study done in Nigeria using different definitions of virological failure found a sensitivity of 66.2% when the cut off similar to the one we used for our study was chosen [8]. Another study comparing the different thresholds for virological failure found that the high threshold is associated with high sensitivity when compared to low threshold [4].

A systematic review that assessed the 2010 WHO immunological criteria found that these criteria have low sensitivity and positive predictive value in identifying individuals with virological failure [12]. The sensitivity and specificity would be expected to be even lower if the ART is initiated at relatively higher CD4 cell count. WHO in 2013 guideline concludes that there is currently no proposed alternative definition of treatment failure and no validated alternative definition of immunological failure [3]. Studies done in children also found low sensitivity and positive predictive value when immunological criteria were used [13, 14]. Based on these facts, WHO recommends routine viral load monitoring (every 6–12 months) to enhance earlier and accurate detection of treatment failure [3]. In settings with limited settings to routine viral load monitoring, WHO recommends targeted viral load strategy to confirm treatment failure diagnosed based on clinical and immunological criteria. Despite the fact that targeted viral load strategy may delay switching to second line and thereby increase the risk of disease progression, it helps to avoid unnecessary switching to second line that could have happened when clinical and immunological criteria were used alone [15].

Our study had several limitations. We could not consider clinical outcomes linked with the treatment failure. Such information is not routinely collected during clinic visits. It was not possible therefore to assess the link between the clinical criteria; we had to focus therefore on laboratory criteria. The study was a cross-sectional study. Longitudinal study design would have been a better design for such a study question. These findings were from a single clinic; the results may not be generalizable to the general population therefore. Our study however is the first study in Tanzania comparing immunological criteria that is the one most used and the gold standard virological criteria in diagnosing treatment failure. It also adds more emphasis on the disadvantages of switching clients to second line based on clinical and immunological criteria that have low sensitivity and positive predictive value; this outlines the need for confirmatory viral load test.

## Conclusion

CD4 criteria for the detection of treatment failure have poor performance for detecting virological failure based on WHO-definition. The low specificity and positive predictive values indicate that using CD4 criteria increases the risk of misclassifications; individuals with adequate virological suppression may be misdiagnosed with treatment failure leading to unnecessary switches while those with virological failure may be missed leading to delay in switching. This further highlights the need to confirm virological failure before switching to second line. Targeted viral load strategy should be used in resource limited settings to confirm treatment failure in cases suspected by clinical and immunological criteria.

## Abbreviations

AIDS: acquired immunodeficiency syndrome
ART: antiretroviral therapy
BMC: Bugando Medical Centre
BMI: body mass index
CD4: cluster of differentiation subtype 4
CTC: care and treatment centre
HIV: human immunodeficiency virus
IQR: interquartile range
NPV: negative predictive value
PPV: positive predictive value
UNAIDS: Joint United Nations Programme on HIV/AIDS
WHO: World Health Organization
